# Oxidative–Inflammatory Crosstalk and Multi-Target Natural Agents: Decoding Diabetic Vascular Complications

**DOI:** 10.3390/cimb47080614

**Published:** 2025-08-04

**Authors:** Jingwen Liu, Kexin Li, Zixin Yi, Saqirile, Changshan Wang, Rui Yang

**Affiliations:** 1 School of Life Science, Inner Mongolia University, Hohhot 010020, China; jwliu200008@163.com (J.L.); kexinli@mail.imu.edu.cn (K.L.); yizixin2001@163.com (Z.Y.); sqrl1009@163.com (S.); 2 Department of Pharmacology, School of Basic Medicine, Inner Mongolia Medical University, Hohhot 010110, China

**Keywords:** diabetes mellitus, vascular complications, oxidative stress, inflammation, natural products, multi-target therapy

## Abstract

Diabetes mellitus (DM) is one of the leading causes of death and disability worldwide and its prevalence continues to rise. Chronic hyperglycemia exposes patients to severe complications. Among these, diabetic vascular lesions are the most destructive. Their primary driver is the synergistic interaction between hyperglycemia-induced oxidative stress and chronic inflammation. This review systematically elucidates how multiple pathological pathways—namely, metabolic dysregulation, mitochondrial dysfunction, endoplasmic reticulum stress, and epigenetic reprogramming—cooperate to drive oxidative stress and inflammatory cascades. Confronting this complex pathological network, natural products, unlike conventional single-target synthetic drugs, exert multi-target synergistic effects, simultaneously modulating several key pathogenic networks. This enables the restoration of redox homeostasis and the suppression of inflammatory responses, thereby improving vascular function and delaying both microvascular and macrovascular disease progression. However, the clinical translation of natural products still faces multiple challenges and requires comprehensive mechanistic studies and rigorous validation to fully realize their therapeutic potential.

## 1. Introduction

Diabetes mellitus is a common metabolic disorder characterized by hyperglycemia, resulting from impaired insulin secretion or action; its prevalence continues to rise. According to International Diabetes Federation data, approximately 537 million people worldwide—10.5% of the adult population—currently live with diabetes, and nearly half remain undiagnosed. By 2045, this number is expected to increase by 46%, to around 783 million [1]. The World Health Organization classifies diabetes into several clinical types, including type 1, type 2, mixed forms, and other specific subtypes. Type 2 diabetes accounts for 90–95% of all cases. Chronic or relative insulin deficiency can lead to serious, multi-organ complications, especially affecting the eyes, kidneys, heart, and vascular system. Diabetic vascular complications—divided into microvascular (retinopathy, nephropathy, and neuropathy) and macrovascular (cardiovascular and cerebrovascular disease) forms—are the most destructive. Their high mortality and disability rates impose a heavy human and economic burden, driving extensive mechanistic research [2,3].

The pathogenesis of diabetic vascular complications depends on an intertwined oxidative–inflammatory network. Chronic hyperglycemia simultaneously activates multiple interconnected pathogenic pathways, including the polyol and hexosamine pathways, protein kinase C (PKC) signaling, and the advanced glycation end products (AGEs)/receptor for advanced glycation end products (RAGE) axis. These disturbances trigger mitochondrial dysfunction, ER stress, and epigenetic reprogramming, collectively driving oxidative stress and inflammatory cascades [4,5].

Current treatment strategies for diabetic vascular complications focus on controlling lipids, blood glucose, and blood pressure through medication, dietary adjustments, and regular exercise. In clinical practice, traditional therapies—such as insulin sensitizers (biguanides and thiazolidinediones) and insulin secretagogues (sulfonylureas and glinides)—effectively lower glycemia but cannot directly reverse this complex stress network [6]. Although novel targeted agents like nuclear factor erythroid 2-related factor 2 (Nrf2) activators, sodium-glucose cotransporter 2 (SGLT2) inhibitors, and glucagon-like peptide-1 (GLP-1) receptor agonists have been developed to overcome the limitations of pure glucose lowering—and have shown localized effects on oxidative–inflammatory nodes in some trials—they remain constrained by a “single-node” approach. They cannot simultaneously address the multiple, interacting pathological pathways present in diabetic vascular disease.

By contrast, natural products (such as polyphenols, terpenoids, and alkaloids) offer multi-target synergy. They can concurrently activate Nrf2-mediated antioxidant defenses, inhibit NF-κB signaling and inflammasome assembly, and modulate epigenetic regulators. As a result, they often provide more pronounced protection—enhancing antioxidant capacity, reducing inflammatory markers, and repairing tissue pathology—than glucose-lowering therapies alone. This multi-faceted approach offers diversified therapeutic options for diabetic vascular complications [7]. However, its limitations are also discussed herein. The clinical translation of many natural products still faces significant obstacles—such as low bioavailability, variable potency, and a lack of large-scale clinical validation—so elucidating their precise mechanisms of action and conducting rigorous, high-quality clinical trials are critical steps toward realizing their therapeutic potential for diabetic vascular complications.

Based on the above, this review systematically elucidates the pathogenesis of diabetic vascular complications and compares the strengths of single-target synthetic drugs with those of multi-target natural products. Our goal is to highlight new translational research directions for the prevention and treatment of diabetic vascular disease.

## 2. Regulatory Networks of Oxidative Stress in Diabetic Vascular Complications

The development of oxidative stress in diabetes involves a sophisticated interplay of multiple interconnected mechanisms. Metabolic pathway dysregulation, mitochondrial dysfunction, endoplasmic reticulum (ER) stress, and epigenetic modulation collectively form the core components of this network: activated metabolic pathways serve as direct responders to hyperglycemia, mitochondria act as primary effectors of oxidative stress, ER stress engages in bidirectional crosstalk with oxidative damage, and epigenetic mechanisms perpetuate the persistent effects of oxidative stress. These elements interact dynamically, creating an intricate regulatory framework that drives the progression of diabetes and its complications. The following sections will detail the molecular basis and functional interplay of these four key dimensions.

### 2.1. Dysregulated Metabolic Pathways

**Figure 1:**
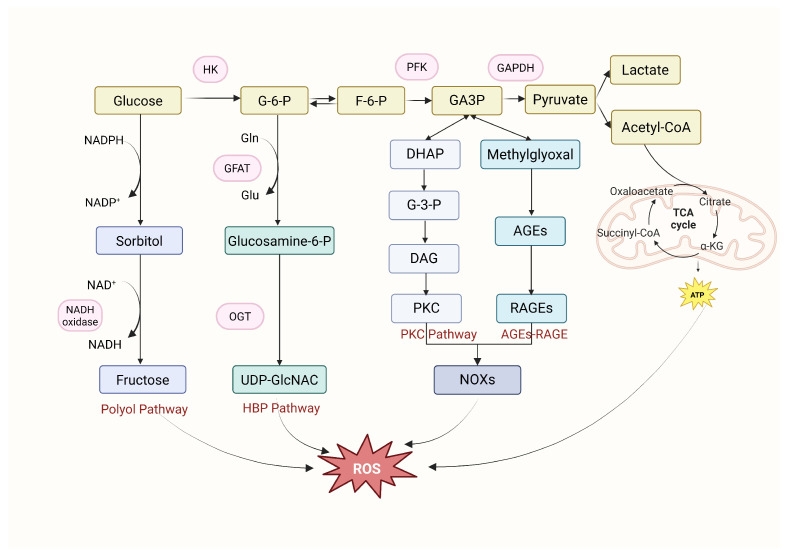
Hyperglycemia-induced metabolic abnormalities in diabetes induce oxidative stress through the activation of 4 pathways (created with BioRender.com). This figure shows how glucose metabolism is diverted into several pathways, including the polyol pathway, activation of the PKC pathway, increased formation of AGEs and increased expression of RAGE, and activation of the hexosamine pathway. These pathways lead to the generation of ROS via different intermediates, such as sorbitol, fructose, DAG, methylglyoxal, and AGE–RAGE interactions. The accumulation of ROS triggers oxidative stress, which plays a key role in the development of diabetic complications. This figure also highlights key enzymes and metabolites involved, including HK, PFK, and pyruvate dehydrogenase, as well as mitochondrial TCA cycle involvement in ATP production. Abbreviations: HK—hexokinase; NADPH—nicotinamide adenine dinucleotide phosphate (reduced form); NADP—nicotinamide adenine dinucleotide phosphate; GFAT—glutamine:fructose-6-phosphate amidotransferase; Glu—glucose; Glucosamine-6-P—glucosamine-6-phosphate; GGP—glucose-6-phosphate; F-6-P—fructose-6-phosphate; PFK—phosphofructokinase; GA3P—glyceraldehyde-3-phosphate; PHAP—phosphohydroxypyruvate; G-3-P—glycerol-3-phosphate; DAG—diacylglycerol; PKC—protein kinase C; NOXs—NADPH oxidases; Acetyl-CoA—acetyl coenzyme A; TCA cycle—tricarboxylic acid cycle; C-KG—α-ketoglutarate; UDP-GlcNAc—uridine diphosphate N-acetylglucosamine; HBP—hexosamine biosynthetic pathway; ROS—reactive oxygen species.

Metabolic abnormalities in diabetes induced by hyperglycemia induce oxidative stress by activating four pathways: activation of the PKC pathway and polyol pathway, increased formation of AGEs, increased expression of RAGE, and activation of the hexosamine pathway (Figure 1). These pathways intricately interact, forming a vicious cycle.

The AGE/RAGE axis is pathologically activated under hyperglycemic conditions, wherein glucose and amino groups undergo non-enzymatic glycation to irreversibly form AGEs. These AGEs exhibit dual prooxidant effects: direct stimulation of reactive oxygen species (ROS) generation and more critically, receptor-mediated activation of NADPH oxidase through RAGE binding, thereby establishing a self-amplifying “AGEs-ROS-AGEs” feed-forward cycle [8,9,10]. This process further activates pivotal signaling pathways including MAPK/ERK and NF-κB, exacerbating oxidative stress and inflammatory responses that ultimately drive the pathogenesis of diverse vascular complications [9].

The pathological activation of the polyol pathway is characterized by enhanced aldose reductase (AR)-mediated glucose conversion, which disrupts the NADH/NAD^+^ ratio and depletes NADPH stores, thereby significantly compromising cellular antioxidant defense capacity [11,12]. As the rate-limiting enzyme of this pathway, AR demonstrates markedly elevated activity in the vascular tissues of diabetic patients, promoting sorbitol accumulation and exacerbating oxidative stress [13,14,15].

The PKC pathway is directly activated through hyperglycemia-induced diacylglycerol (DAG) accumulation, while being concurrently modulated by both AGE/RAGE signaling and polyol pathway activity [16,17]. Activated PKC disrupts vascular homeostasis by suppressing NOS-derived vasodilation while enhancing endothelin-1-mediated vasoconstriction, ultimately driving fibrotic and inflammatory vascular remodeling [18,19,20].

The hexosamine biosynthetic pathway plays a pivotal role in regulating glucose metabolism and insulin sensitivity. This metabolic pathway converts glucose-6-phosphate into uridine diphosphate N-acetylglucosamine (UDP-GlcNAc), who’s derivative GlcNAc mediates kinase-like serine/threonine phosphorylation, which inhibits AKT (protein kinase B)/eNOS phosphorylation. This post-translational modification subsequently enhances the expression of both transforming growth factor-β (TGF-β) and plasminogen activator inhibitor-1 (PAI-1), ultimately contributing to oxidative stress and fibrotic progression [21,22].

### 2.2. Mitochondrial Dysfunction

Persistent hyperglycemia induces mitochondrial dysfunction and triggers oxidative stress through multiple mechanisms [23]. Under chronic hyperglycemic conditions, the mitochondrial electron transport system becomes functionally compromised, characterized by increased electron leakage primarily from complexes I, III, and II of the electron transport chain (ETC). This pathological electron escape results in excessive generation of superoxide radicals (O_2_^−^), a primary ROS [24]. Concurrently, compromised functionality of key intracellular antioxidant defense systems—specifically superoxide dismutase (SOD), glutathione (GSH), and catalase (CAT)—disrupts redox homeostasis, further aggravating ROS accumulation [25]. This metabolic dysregulation manifests during early-stage diabetes as elevated complex I/II-dependent oxidative phosphorylation (OXPHOS) activity in hepatic mitochondria, which progressively declines with disease progression, ultimately resulting in impaired ATP synthesis and an energy metabolism crisis [26,27]. At the organelle level, chronic hyperglycemia disrupts mitochondrial homeostasis by upregulating the pro-fission protein dynamin-related protein 1 (Drp1) while downregulating the fusion regulator mitofusin 2 (Mfn2), ultimately leading to mitochondrial fragmentation [28,29]. Moreover, impaired PTEN-induced putative kinase 1 (PINK1)/Parkin-mediated mitophagy exacerbates the accumulation of dysfunctional mitochondria [30]. Persistent ROS accumulation induces mitochondrial membrane potential (ΔΨm) collapse and triggers the release of pro-apoptotic factors (e.g., cytochrome c), ultimately accelerating β-cell apoptosis and vascular damage [31,32]; furthermore, it may impair mitochondrial biogenesis by suppressing key regulatory factors such as peroxisome proliferator-activated receptor-γ coactivator-1α (PGC-1α) [30,33], and it concurrently activates PKC and facilitates the formation of AGEs, directly contributing to vascular complications [34]. Notably, certain hypoglycemic agents (e.g., metformin and SGLT2 inhibitors) may indirectly improve mitochondrial function by modulating electron transport chain (ETC) activity or enhancing antioxidant defenses, suggesting that targeting mitochondrial metabolism represents a potential therapeutic strategy for alleviating diabetic oxidative stress [26,28].

### 2.3. Endoplasmic Reticulum Stress

Endoplasmic reticulum (ER) serves as a significant intracellular source of ROS, accounting for 25% of total cellular ROS generation through oxidative protein folding [35]. During disulfide bond formation, ER oxidoreductase 1 (ERO1) catalyzes the re-oxidation of protein disulfide isomerase (PDI). This reaction uses molecular oxygen as an electron acceptor, generating hydrogen peroxide (H_2_O_2_) as a byproduct [36,37]. This ROS production is counterbalanced by ER-resident antioxidants, including peroxiredoxin 4 (PRDX4) and glutathione peroxidases 7/8 (GPX7/GPX8) [38,39,40].

When ROS accumulation exceeds ER’s antioxidant capacity, it induces ER stress and activates the unfolded protein response (UPR) through three major signaling branches [41]. The PERK pathway phosphorylates eukaryotic initiation factor 2α (eIF2α) to attenuate global translation while selectively promoting activating transcription factor 4 (ATF4) translation, which upregulates antioxidant response elements [42,43,44]. The inositol-requiring enzyme 1 (IRE1) pathway exhibits dual functions: its kinase domain activates JNK signaling through TNF receptor-associated factor 2 (TRAF2) recruitment; concurrently, its RNase activity mediates regulated IRE1-dependent decay (RIDD) of microRNAs, including miR-17. This RIDD activity leads to thioredoxin-interacting protein (TXNIP) upregulation and subsequent NLRP3 inflammasome activation [45,46,47]. The activating transcription factor 6 (ATF6) pathway undergoes proteolytic activation in the Golgi apparatus to modulate the expression of ER quality control proteins [48].

Notably, these pathways establish a vicious cycle with oxidative stress. ROS activates calcium release channels on the ER membrane. These channels include inositol trisphosphate receptors (IP3Rs) and ryanodine receptors (RyRs). Released calcium ions are taken up by mitochondria at mitochondria-associated membranes (MAMs). This process further stimulates ROS production [49]. Additionally, the UPR effector CHOP exacerbates oxidative stress by upregulating ERO1 expression, creating a feed-forward loop of H_2_O_2_ generation [41]. This intricate crosstalk between oxidative stress and ER stress fundamentally contributes to cellular homeostasis dysregulation under pathological conditions.

### 2.4. Epigenetic Regulation

Epigenetic modifications serve as both early targets of hyperglycemia and amplifiers of persistent oxidative stress. The hyperglycemic milieu induces epigenetic reprogramming through DNA methylation, histone modifications, and altered non-coding RNA expression, establishing a self-reinforcing pathological cycle. These modifications not only directly regulate the expression of oxidative stress-related genes but also perpetuate abnormal oxidative stress states through “metabolic memory” effects [50], which persist even after achieving strict glycemic control. The epigenetic mechanisms participate in oxidative stress regulation through multiple tiers: DNA methylation alters the expression balance between antioxidant and pro-inflammatory genes, histone modifications dynamically modulate the amplification of oxidative stress signals, and ncRNA networks precisely regulate redox homeostasis-related pathways [51]. This vicious cycle between epigenetic alterations and oxidative stress constitutes a core mechanism underlying the pathogenesis of diabetic vascular complications.

#### 2.4.1. DNA Methylation

Hyperglycemia can induce DNA methylation modifications that affect redox homeostasis. Studies demonstrate that hyperglycemia reduces the expression of the methyltransferase DNMT3b, leading to CpG hypomethylation at the promoter region of the pro-oxidant adaptor protein p66Shc, thereby enhancing its transcription and exacerbating oxidative stress [52]. Moreover, excessive ROS production can induce aberrant methylation of genes such as TGF-β1, further driving disease progression [53]. DNA methylation may also regulate the expression of Nrf2, highlighting the potential therapeutic utility of demethylating agents [54]. Concurrently, DNA methylation modulates NAD^+^ metabolism through key enzymatic regulation [55]. Specifically, nicotinamide N-methyltransferase (NNMT) catalyzes the methylation of nicotinamide (NAM) to 1-methylnicotinamide (MNA), thereby preventing NAM from entering the NAD^+^ salvage pathway. Methylation of NAM by NNMT reduces its intracellular concentration; conversely, high levels of NAM inhibit the activity of SIRT1, an NAD^+^-dependent deacetylase [56,57,58]. Inhibition of NNMT activity elevates intracellular NAD^+^ levels and modulates NAD^+^-dependent enzymatic functions [59,60,61]. Under hyperglycemic conditions, DNA damage activates poly (ADP-ribose) polymerase 1 (PARP1), depleting NAD^+^ pools and inhibiting SIRT1 activity. This process establishes a pathological cycle of “NAD^+^ depletion–SIRT1 inhibition–oxidative stress” [62,63,64,65,66]. These epigenetic alterations persist following glycemic normalization, sustaining a state of NAD^+^ redox imbalance that underlies diabetic vascular complications [60,67].

#### 2.4.2. Histone Modifications

Histone modifications are crucial epigenetic mechanisms, encompassing methylation, acetylation, and other post-translational modifications [68]. Histone acetylation (HAc) is intricately associated with oxidative stress [69,70]. Activation of the histone acetyltransferases (HATs) p300/CBP increases ROS production by upregulating NADPH oxidase and inflammatory mediators [71]. SIRT1 deacetylates peroxisome PGC-1α, enhancing mitochondrial biogenesis and protecting against metabolic stress [72,73]. SIRT1 maintains energy homeostasis by directly stimulating mitochondrial fatty acid β-oxidation genes and suppresses NF-κB signaling through deacetylation, attenuating inflammation in metabolic disorders [74,75]. Resveratrol, functioning as a SIRT1 agonist, effectively reduces excessive ROS generation under hyperglycemic conditions [76]. Valproate, acting as a histone deacetylase (HDAC) inhibitor, suppresses the NF-κB/iNOS signaling pathway [77].

#### 2.4.3. Non-Coding RNAs

Non-coding RNAs (ncRNAs), including microRNAs (miRNAs), long ncRNAs (lncRNAs), and circular RNAs (circRNAs), play critical roles in oxidative stress, inflammation, and apoptosis. Experimental evidence confirms that miR-218 and miR-34a function as upstream regulators of p66Shc, modulating DNA/histone modification networks to sustain p66Shc expression and oxidative stress in the hearts of diabetic mice [78,79]. MiR-218 exacerbates high-glucose-induced apoptosis by activating the p38 MAPK pathway [79]. In contrast, miR-34a inhibition attenuates cardiomyocyte death and fibrosis, thereby improving cardiac function [78]. NF-κB downregulates SIRT1 activity via miR-34a, establishing a pro-inflammatory loop in diabetic vasculature [75]. Sustained miR-34a expression in diabetes accelerates endothelial dysfunction by repressing SIRT1-mediated antioxidant responses [75,80]. Furthermore, miR-485 mitigates high glucose-induced ROS production by targeting Nox5 while upregulating SOD activity [81]. LncRNAs (e.g., NEAT1) operate as molecular sponges for miRNAs, modulating target gene expression to influence proliferation, oxidative stress, inflammation, and fibrosis [82,83]. Similarly, circRNAs (e.g., circHOMER1) enhance oxidative stress and inflammatory responses via the miR-137/SOX6 axis [84]. These findings position ncRNAs as promising therapeutic targets for metabolic cardiovascular diseases.

## 3. Regulatory Networks of Inflammatory Response in Diabetic Vascular Complications

Chronic inflammation serves as a central driver of diabetic vasculopathy, in which systemic inflammatory processes create a permissive pathological environment and localized vascular responses determine tissue-specific injury patterns. This section examines the interplay between fundamental triggers of systemic inflammation and the specialized mediators that drive vascular complications.

### 3.1. Systemic Inflammation in Diabetes

Inflammation plays a crucial role in numerous physiological and pathological processes, especially in chronic diseases such as diabetes [85,86,87]. The initiation of the inflammatory process is typically associated with infection, tissue injury, or immune dysregulation, leading to the recruitment of leukocytes and the release of inflammatory cytokines in the affected tissues [88,89]. Major pro-inflammatory mediators include tumor necrosis factor-α (TNF-α), interleukin-1β (IL-1β), and interferons such as IFN-γ, as well as the transcription factor NF-κB [90]. In contrast, key anti-inflammatory cytokines include IL-1 receptor antagonist (IL-1RA), IL-4, IL-10, IL-11, and IL-13 [91]. All of these mediators participate in the regulation of the human immune response.

**Figure 2:**
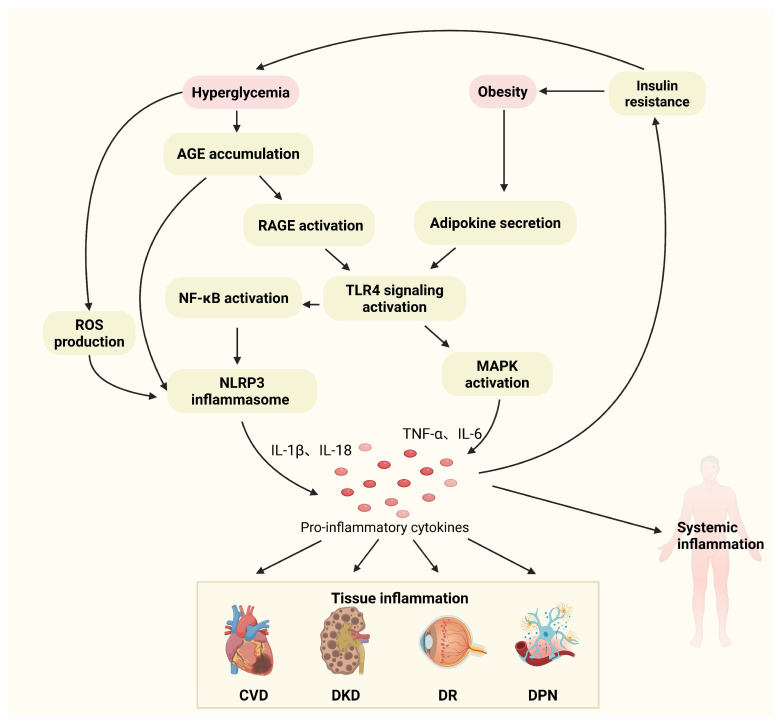
Molecular mechanisms underlying inflammatory responses in diabetes (created with BioRender.com). This figure illustrates how hyperglycemia and obesity synergistically activate the NLRP3 inflammasome via the AGE–RAGE axis, adipokine secretion, and TLR4/NF-κB signaling. This activation results in IL-1β/IL-18 release and MAPK-mediated amplification of pro-inflammatory cytokines (e.g., TNF-α, IL-6). These processes culminate in tissue inflammation and diabetic vascular complications. NLRP3 inflammasome assembly requires dual signals: metabolic stress (ROS/AGEs) as the triggering signal, and TLR4/NF-κB as the priming signal. Abbreviations: AGE—advanced glycation end products; RAGE—receptor for advanced glycation end products; TLR4—Toll-like receptor 4; NLRP3—NLR family pyrin domain-containing 3; IL-1β—interleukin-1 beta; TNF-α—tumor necrosis factor alpha; IL-6—interleukin-6; CVD—cardiovascular disease; DKD—diabetic kidney disease; DR—diabetic retinopathy; DPN—diabetic peripheral neuropathy; ROS—reactive oxygen species; NF-κB—nuclear factor kappa B.

At present, it is believed that diabetes is closely related to chronic inflammation in the whole body [92]. Circulating levels of inflammatory markers—such as pro-inflammatory cytokines, chemokines, adhesion molecules, and transcription factors—are significantly elevated in patients with diabetes, and these factors contribute to both the onset and progression of the disease [93]. In type 2 diabetes mellitus (T2DM), chronic inflammation damages the pancreatic β-cells leading to insufficient insulin secretion and subsequent hyperglycemia [94]. Alterations in circulating and urinary mitochondrial DNA are also correlated with inflammation in T2DM [95]. Moreover, inflammation also induces hepatic insulin resistance [96]. The proinflammatory cytokine TNF-α, a mediator of insulin resistance, regulates insulin resistance in T2DM and other metabolic diseases [97]. IL-1β regulates the immune and inflammatory response in T2DM [98]. In addition, studies have shown that the NLRP3 inflammasome plays vital roles in the development of diabetes [99]. The specifically pro-inflammatory cytokines and other molecules are also critical factors in the development of vascular diabetic complications [100]. As shown in Figure 2, hyperglycemia and obesity promote the development of diabetic vascular complications by activating dual pathways—TLR4/NF-κB signaling and the NLRP3 inflammasome—which drive the release of pro-inflammatory factors [101]. The following sections will provide detailed analysis of the regulatory roles of key inflammatory factors.

### 3.2. Vascular Inflammatory Mediators

The inflammation process is regulated by a variety of inflammatory cytokines: pro-inflammatory factors such as TNF-α, IL-1β, IL-6, and IL-8; the adhesion factors intercellular adhesion molecule 1 (ICAM-1) and vascular cell adhesion molecule-1 (VCAM-1); and some other inflammatory factors, such as monocyte chemotactic protein 1 (MCP-1), NF-kB, NLRP3, and so on [102,103,104]. Dysregulation of these factors occurs during the development of diabetic complications. In this review, we focus on recent studies and novel mechanisms underlying these processes.

#### 3.2.1. Pro-Inflammatory Factors

TNF-α is both a biomarker and therapeutic target for diabetic complications [105,106,107]. Elevated circulating TNF-α has been used as a biomarker for diabetic kidney disease (DKD). As reported, local TNF-α, other than serum TNF-α, is sufficient to induce podocyte injury that depends on free cholesterol. This injury is mediated through an NFATc1/ABCA1-dependent mechanism [108]. The tear levels of IL-1RA, IL-8, and TNF-α could reflect diabetes and its progression in severity [109]. Inactivation of TNF-α ameliorates diabetic neuropathy in mice [110,111], and inhibition of IL-1β significantly alleviates diabetic nephropathy [112].

#### 3.2.2. Adhesion Factors

The ICAM-1 signaling pathway is activated in diabetic retinopathy, and increased levels of ICAM-1 are associated with the severity of DR [113,114]. Downregulation of the expression of VEGF and ICAM-1 could protect against diabetic retinopathy [115]. Levels of VCAM-1 are significantly associated with microvascular complications of diabetes [116], while elevated ICAM-1 and MCP-1 plasma levels imply high cardiovascular risk [117].

#### 3.2.3. Other Inflammatory Factors

The NLRP3 inflammasome plays an important role in the development and progression of diabetic retinopathy [118]. Activation of the NLRP3 inflammasome contributes to diabetic nephropathy’s development [119], while inhibition of the NLRP3 inflammasome alleviates vascular calcification in diabetic nephropathy [120]. NLRP3 is also involved in pyroptosis in diabetic complications [121,122]. Podocyte injury in murine models of diabetic nephropathy is associated with NLRP3-induced inflammation and pyroptosis [123]. The NLRP3 inflammasome is also a therapeutic target of diabetic complications. Inhibiting NLRP3 could protect against insulin resistance and myocardial fibrosis in diabetic cardiomyopathy (DCM) models [124].

## 4. Oxidative Stress in Diabetic Vascular Complications

DM precipitates various complications, among which vascular disorders affecting both macrovascular and microvascular systems pose the most severe threats to human health and socioeconomic wellbeing [125]. The pathogenesis centers on chronic oxidative stress—an imbalance between ROS/RNS overproduction and compromised antioxidant defenses (SOD, GPX, and vitamins) [126,127]. This oxidative state damages cellular components and mitochondria while triggering inflammatory cascades through NF-κB activation [128,129,130,131,132,133]. Clinical studies confirm elevated oxidative stress in diabetics, where persistent hyperglycemia and mitochondrial dysfunction drive ROS accumulation while impairing radical scavenging capacity [134,135,136,137,138]. The consequences are threefold: metabolic deterioration through β-cell dysfunction and insulin resistance [136,139,140], direct biomolecular damage [141], and accelerated vascular injury—manifesting as retinopathy, nephropathy, neuropathy, and cardiovascular disease—through oxidative–inflammatory crosstalk mediated by the ICAM-1 and NLRP3 inflammasomes [142,143]. Thus, a self-amplifying cycle arises: hyperglycemia induces oxidative stress, which fuels tissue damage and metabolic decline, highlighting a pivotal therapeutic target.

### 4.1. Retinopathy

Diabetic retinopathy (DR) is caused by chronically high blood glucose damaging blood vessels in the retina, accompanied by retinal neurodegeneration in the early stages [144], involving changes in vascular permeability, capillary microaneurysms, capillary degeneration, and excessive formation of new blood vessels.

Oxidative stress plays a pivotal role in the pathogenesis and progression of DR. The retinal pigment epithelium (RPE) is vulnerable to oxidative damage [145], since photoreceptors are the main source of ROS in the retina. Mitochondrial dysfunction in the RPE increases the production of ROS and induces oxidative stress in the retina [146]. Oxidative stress in RPE cells then injures them by promoting pro-inflammatory gene expression and disrupting cell proliferation [147]. Oxidative stress in retinal microvascular endothelial cells reduces the expression of hypoxia-inducible factor alpha (HIF1α), which subsequently upregulates vascular endothelial growth factor (VEGF), promoting angiogenesis and vascular leakage. Additionally, oxidative stress enhances retinal inflammation by increasing the expression of NF-κB, MCP-1, and ICAM-1 [148]. Activation of oxidative stress can also cause mitochondrial dysfunction and promote the apoptosis of human retinal endothelial and RPE cells [149,150]. Furthermore, oxidative stress inhibits the Nrf2 antioxidant response element (ARE) pathway in the retina, exacerbating tissue injury [151]. The p38-MAPK pathway is also activated under oxidative stress in DR, suggesting that p38-MAPK inhibition may prevent oxidative damage [152,153,154]. Finally, activation of aldo-keto reductase family 1 member B1 (AKR1B1), the first enzyme in the polyol pathway, promotes DR under oxidative stress; therefore, inhibition of AKR1B1 represents a promising therapeutic strategy [155].

### 4.2. Nephropathy

Diabetic nephropathy (diabetic kidney disease (DKD)) is one of the leading causes of all chronic kidney diseases and end-stage renal disease [156,157]. The pathophysiology of DKD is complicated. Hemodynamic and metabolic dysfunction induced by hyperglycemia can increase the glomerular filtration rate and glomerular volume [158]. Inflammation and oxidative stress are also recognized as major drivers of DKD progression [159,160,161]. Moreover, activation of the renin–angiotensin–aldosterone system (RAAS) is associated with the pathogenesis of DKD [162]. Additionally, ER stress is a major contributor to DKD [163]. In type 2 diabetes, chronic hyperglycemia and glomerular hyperfiltration lead to glomerular, proximal tubular, and mitochondrial dysfunction [164]. Dysfunction of mitochondria and NADPH oxidase represents major sources of oxidants, which significantly increase the risk of DKD [165]. Angiotensin II (Ang II) also promotes podocyte injury in DKD by enhancing calcium influx and the generation of ROS [166]. Glycation of some proteins also plays a crucial role in the development and progression of DKD; for example, glycated albumin promotes kidney fibrosis [167]. Understanding DKD’s pathogenesis is crucial for developing new therapeutic approaches.

Oxidative stress holds a key position in the pathogenesis of chronic kidney disease, including DKD [168]; it plays a critical role in the initiation and progression of DKD [169]. The kidneys are rich in oxidation reactions in mitochondria, making them more vulnerable to oxidative stress [170]. Chronic stimulation of hyperfiltration and hyperexcretion in the tubules leads to relative ischemia, local hypoxia, and the loss of nephrons [171], causing the accumulation of ROS, and later activating oxidative stress. This can damage podocytes, mesangial cells, and endothelial cells, resulting in proteinuria and tubulointerstitial fibrosis [157,172,173]. Excess amounts of ROS modulate the activation of PKC, mitogen-activated protein kinases, and various cytokines and transcription factors [174]. In DKD, accumulation of ROS in the kidney is implicated in renal inflammation, affecting renal structure and function [175,176]. Studies show that NADPH oxidases (NOX), particularly the Nox4 and Nox5 isoenzymes, are the primary contributors to ROS production in diabetic kidneys [177]. Nox4 is a NOX isoenzyme that is highly expressed in the kidneys, and its expression level is significantly increased under hyperglycemic conditions. Nox4 functions by generating H_2_O_2_, which plays a crucial role in maintaining certain physiological functions of the kidneys [178]. Nox4 is present in renal tubular epithelial cells, glomerular mesangial cells, and endothelial cells, and it plays a key role in renal oxidative stress in DKD, making it a potential therapeutic target for inflammatory kidney diseases [179]. Nox5 is present in glomeruli, and in diabetic kidneys its expression is upregulated, making it a major source of renal ROS. Silencing Nox5 reduces ROS production and alleviates inflammation and fibrosis induced by diabetes [180]. Therefore, inhibitors targeting Nox4 and Nox5 are considered to be potential drug targets for the treatment of diabetes-related kidney disease [181].

### 4.3. Neuropathy

Diabetic peripheral neuropathy (DPN) manifests as progressive peripheral nerve dysfunction advancing from distal to proximal regions [182]. The pathogenesis of DPN is complicated, involving both the somatic and autonomic divisions of the peripheral nervous system, comprising both sensory and motor neurons. Hyperglycemia is a risk factor for DPN [183]. As reported previously, hyperglycemia directly injures sensory neurons in the dorsal root ganglia (DRG), leading to distal axonal degeneration [184].

Enhanced cellular oxidative stress is also a vital initiator of DPN [185]. The source of the ROS in DPN is mainly through the pathways mentioned above, including the activated polyol pathway, hexosamine pathway, etc. [186]. Hyperglycemia activates the polyol pathway by depleting NADPH, leading to increased oxidative stress and impaired glutathione regeneration. In DPN, sorbitol and fructose accumulate in the peripheral nerves of diabetic rats, and the diversion of glycolytic intermediates to the polyol pathway exacerbates glycation [187]. Inhibiting aldose reductase can prevent this accumulation; therefore, inhibiting the polyol pathway is an effective therapeutic target for DPN [185]. The mechanism by which the hexosamine pathway contributes to DPN involves the impact of intracellular UDP-GlcNAc on protein modification [188]. Hyperglycemia-induced over-modification of proteins by glucosamine leads to pathological changes in gene expression, particularly in transcription factors, which play a critical role in the pathogenesis of diabetic complications [189]. Simultaneously, oxidative stress not only activates those major pathways but also initiates and amplifies neuro-inflammation [190]. In DPN, sensory neurons in the DRG are attacked by hyperglycemia and metabolic stressors, leading to distal axonal degeneration [184]. ROS accumulated in the DRG and peripheral nerves activate several pathways—including mitochondrial function, ER stress, autophagy, and epigenetic signaling—to promote the progression of DPN [191]. Meanwhile, another study pointed out that ROS production in the injured DRG may also be necessary for axonal regeneration [192]. Thus, the role of oxidative stress and ROS accumulation in DRG during DPN remains to be elucidated.

### 4.4. Cardiovascular Disease

Cardiovascular disease (CVD) is a leading cause of death for patients with DM. Cardiovascular diseases in diabetic patients mainly include atherosclerotic cardiovascular disease (ASCVD) and DCM, among which ASCVD includes coronary heart disease, cerebrovascular disease and peripheral vascular disease. Diabetes mellitus can induce the development of atherosclerosis or accelerate its progression, which is the underlying cause of most myocardial infarctions and stroke [193,194]. Endothelial dysfunction, inflammation, and oxidative stress are hallmarks of atherosclerosis [195]. Diabetic cardiomyopathy is characterized by diastolic relaxation abnormalities in the early stages and, later, heart failure occurring without coronary artery disease, hypertension, and dyslipidemia [196,197].

The development of diabetic atherosclerosis and diabetic cardiomyopathy is strongly associated with oxidative stress. Atherosclerosis develops in disturbed vascular regions undergoing oxidative stress [198]. Vascular oxidative stress then facilitates the oxidative modification of lipoproteins and phospholipids, impairs endothelial function, and activates inflammation [136,199,200]. Oxidative stress can also accelerate the development of atherosclerosis by activating the ubiquitin proteasome system, inhibiting the activation of AMPK and adiponectin and decreasing endothelial nitric oxide synthase activity [201,202]. Moreover, ROS can also inactivate the effect of anti-atherosclerosis enzymes in diabetic atherosclerosis [203]. As the main source of ROS, NOx enzymes are inevitably intertwined with the pathogenesis of atherosclerosis. However, unlike complications such as diabetic nephropathy, Nox4 has been shown to have a protective effect against atherosclerosis [204]. In a hyperglycemic environment, cardiomyocytes increase flux through oxidative stress pathways. The flux changes through these pathways may lead to the generation of mitochondrial ROS, non-enzymatic protein glycation, and glucose auto-oxidation [205]. Accumulation of ROS in cardiomyocytes is a leading cause of severe cardiac remodeling by inducing mitochondrial dysfunction and lipid peroxidation. Moreover, oxidative stress can also affect various kinds of signaling pathways to promote the development of diabetic cardiomyopathy [206]. For example, secreted frizzled-related protein 2 (SFRP2) reduced oxidative stress in DCM by regulating AMPK-PGC1 signaling and then exerted cardioprotective effects [207], indicating that targeting oxidative stress and its relative signaling pathways is a reliable way to find new approaches in the treatment of DCM.

## 5. Antioxidant and Anti-Inflammatory Therapies for Diabetic Vascular Complications

Treatment for diabetic vascular complications has always been limited, and studying the pathogenesis process of those complications has revealed new targets for treatment. Here, we summarize some new perspectives on antioxidant and anti-inflammation therapy.

### 5.1. Synthetic Drugs Targeting Oxidative Stress and Inflammation

The pathological progression of diabetic vascular complications is closely associated with oxidative stress and chronic inflammation. In clinical practice, synthetic drugs have become pivotal therapeutic agents due to their well-defined target specificity and modifiable pharmacokinetic profiles. This section focuses on four clinically validated synthetic drug classes: Nrf2 activators alleviate oxidative damage by enhancing antioxidant defenses, SGLT2 inhibitors and GLP-1 receptor agonists cooperatively regulate metabolism-associated oxidative stress through pleiotropic mechanisms, and XO inhibitors directly block purine metabolism-derived inflammatory cascades. These agents exert therapeutic effects by precisely targeting core molecular pathways in diabetic complications, with GLP-1 receptor agonists demonstrating unique gut–vascular axis protection. Notably, this discussion is confined to chemically synthesized or structurally optimized clinical drugs, whereas naturally derived agents will be detailed in Section 5.2.

#### 5.1.1. Nrf2 Activators

Nrf2 is a transcription factor that primarily regulates the expression of genes activated in response to elevated levels of ROS, thereby facilitating the elimination of ROS accumulation. Under normal physiological conditions, Nrf2 is sequestered in the cytoplasm by Keap1 and undergoes ubiquitination followed by proteasomal degradation. However, under oxidative stress, Nrf2 translocates into the nucleus, where it promotes the expression of antioxidant genes [208]. In Nrf2-deficient mice, reduced expression of antioxidant genes leads to increased oxidative stress and the activation of pro-inflammatory signals [209]. Nrf2 exerts its anti-inflammatory effects by interacting with NF-κB and directly inhibiting the transcription of the cytokines IL-1β and IL-18 [210]. Due to its role in oxidative stress and anti-inflammatory processes, Nrf2 is a therapeutic target for diabetic vascular complications. Synthetic Nrf2 agonists, exemplified by bardoxolone methyl, activate this pathway by inhibiting Keap1’s function [211]. In a recent 52-week clinical trial, bardoxolone methyl significantly improved renal function in patients with type 2 diabetes and comorbid chronic kidney disease (CKD) [212]. However, early-phase studies indicated that bardoxolone methyl elevated the risk of early-onset fluid overload in patients harboring identifiable risk factors for heart failure [213]. Beyond Keap1 inhibition, alternative activation strategies—including transcriptional activation of the Nfe2l2 gene, degradation of Keap1 mRNA, and blockade of Nrf2 proteasomal degradation—demonstrate therapeutic potential for preventing diabetic vascular complications [214].

#### 5.1.2. SGLT2 Inhibitors and GLP-1RAs

Studies have shown that SGLT2 inhibitors improve hyperglycemia-induced vascular dysfunction by alleviating oxidative stress and inflammation through their glucose-lowering effects, as well as restoring insulin signaling [215]; they also prevent oxidative stress, AGE signaling, and inflammation by inhibiting NADPH oxidase (NOX) [216]. This provides multiple benefits by inducing systemic and glomerular hemodynamic changes, offering metabolic advantages, and reducing inflammation and oxidative stress pathways [217,218,219]. In addition, studies have found that SGLT2 is also expressed in the human retina, and it plays a crucial role in the prevention and treatment of diabetic retinopathy by improving pathogenic factors of diabetes, protecting the blood–retinal barrier (BRB), and preserving the optic nerve, among other mechanisms [220]. These studies highlight the immense therapeutic potential of SGLT2 inhibitors in the treatment of diabetes. Notably, SGLT2 inhibitors carry clinically significant risks, including class-wide adverse effects such as diabetic ketoacidosis (DKA) or urinary and genital infections, as well as drug-specific complications—exemplified by increased bladder cancer incidence with dapagliflozin and elevated amputation/fracture risks with canagliflozin [6,221].

GLP-1 receptor agonists (GLP-1RAs), such as liraglutide and semaglutide, ameliorate diabetic vascular complications through pleiotropic mechanisms [222]. Studies demonstrate that GLP-1RAs activate the AMPK-SIRT1 pathway to enhance antioxidant defenses while suppressing NOX4-mediated ROS generation, thereby mitigating oxidative stress-induced damage [223]. Their anti-inflammatory actions are characterized by the inhibition of NF-κB signaling and NLRP3 inflammasome activation, significantly reducing pro-inflammatory cytokines such as IL-6 [224]. Clinical evidence confirms that liraglutide therapy significantly reduces major adverse cardiovascular events and slows the deterioration of renal function in patients with type 2 diabetes [10]. These protective effects occur partially independent of glycemic control, indicating direct vasculoprotective properties of GLP-1RAs. However, GLP-1 receptor agonists are associated with transient gastrointestinal disturbances (notably nausea and diarrhea), injection site reactions, including localized irritation and nodule formation, and pancreatitis [225]; moreover, their use may pose additional risks in patients with renal impairment or end-stage renal disease (ESRD) [6,226].

#### 5.1.3. XO Inhibitors

Xanthine oxidase (XO) is a key enzyme in purine metabolism, and hyperglycemia abnormally activates this protein in the kidneys of diabetic rats. Highly activated XO increases intracellular ROS levels, causing kidney damage by directly oxidizing renal cells and indirectly inducing inflammatory responses through the activation of the NF-κB signaling pathway [227]. XO inhibitors, including allopurinol, are a group of anti-inflammatory compounds. Studies have shown that XO inhibitors exert a protective effect against DKD, primarily mediated by their anti-inflammatory properties [228]. This anti-inflammatory property expands the therapeutic potential of XO inhibitors for other diabetic complications. Oral administration of allopurinol, which inhibits xanthine oxidoreductase (including xanthine oxidase and xanthine dehydrogenase), can reduce the levels of inflammatory cytokines [229]. Notably, clinical evidence has established allopurinol hypersensitivity syndrome (AHS) as a rare yet life-threatening adverse reaction associated with allopurinol therapy, necessitating vigilant monitoring [230].

### 5.2. Synergistic Modulation of Oxidative Stress and Inflammation by Natural Compounds

Diverging from the single pathway-targeted synthetic drugs discussed in Section 5.1, natural compounds confer unique therapeutic advantages through multicomponent synergy. Plant-derived secondary metabolites—including polyphenols, flavonoids, terpenoids, and alkaloids (Table 1) —concurrently modulate core signaling networks (e.g., AMPK/SIRT1, Nrf2, and NF-κB) to potentiate antioxidant and anti-inflammatory effects with minimal toxicity. Emerging evidence further demonstrates that several natural compounds can directly modulate epigenetic regulators to reprogram gene expression and sustain their antioxidant and anti-inflammatory benefits. Compared to the high specificity of synthetic agents, the multi-target nature of natural compounds enables more comprehensive intervention in the pathological cascades of diabetic vascular complications, offering novel avenues for developing high-efficacy, low-toxicity therapies [6]. However, the clinical application of these compounds is frequently hampered by poor bioavailability, inconsistent potency, and patient heterogeneity.

#### 5.2.1. Polyphenols

Polyphenols are a class of bioactive compounds with multiple phenolic ring structures; they are the most common antioxidants in the human diet and are present in fruits, vegetables, and products made from them, such as cereals and beverages [231,232]. These secondary metabolites are classified as phenolic acids, flavonoids, or non-flavonoids, according to their chemical structure [233]. Flavonoids, while technically a subclass of polyphenols, are highlighted here separately due to their pronounced therapeutic importance in the management of diabetic complications. Polyphenols are considered to have antioxidant, antibacterial, anti-inflammatory, anticancer and antidiabetic effects. They are beneficial to health and are the most abundant compounds in the diet. Because of their antioxidant, anti-inflammatory, and antidiabetic properties, they are considered to show effective therapeutic potential in the management of diabetic complications [232].

Resveratrol is a plant-derived non-flavonoid polyphenolic compound that is mainly found in cereals, fruits, vegetables, dried beans, and plant-derived beverages [234,235]. Resveratrol exhibits diverse biological and pharmacological properties, including anti-obesity, antidiabetic, anticancer, anti-inflammatory, antioxidant, and cardiovascular-protective effects [234]. Recent studies have demonstrated that resveratrol supplementation may prevent diabetes mellitus and its cardiovascular complications [235]. The protective effects of resveratrol involve the modulation of multiple signaling pathways. In terms of its antioxidant properties, resveratrol directly scavenges ROS. It also activates the SIRT1/FOXO pathway, which enhances the expression of antioxidant genes [236]. Concurrently, resveratrol inhibits NADPH oxidase (NOX2/4), thereby reducing ROS generation. In addition, it regulates the activity of SOD via the AMPK pathway. Resveratrol further activates the Nrf2 pathway, leading to the induction of antioxidant enzymes such as heme oxygenase-1 (HO-1). Through these mechanisms, resveratrol effectively mitigates oxidative damage induced by hyperglycemia [234,237,238,239]. Regarding its anti-inflammatory mechanisms, AMPK-dependent promotion of Nrf2 nuclear translocation concurrently reduces pro-inflammatory cytokine production and establishes potent antioxidant–anti-inflammatory crosstalk [234,240]; furthermore, it inhibits the NF-κB signaling pathway to reduce inflammatory mediator release, effectively alleviating inflammatory responses in diabetic complications [241]. Although studies indicate that oral RES supplementation exerts beneficial effects on glomerular function by modulating inflammatory factors in patients with overt type 2 diabetic nephropathy, its clinical application in diabetic patients requires further investigation through controlled, large-scale clinical trials [242].

Curcumin is a yellow polyphenolic bioactive compound derived from the rhizomes of *Curcuma longa* L. (Zingiberaceae) [243]. Curcumin exhibits a broad spectrum of pharmacological activities, including antitumor, anti-inflammatory, antioxidant, immunomodulatory, anti-ischemic, antithrombotic, hepatoprotective, hypolipidemic, and analgesic effects [244,245]. Notably, large-scale randomized controlled trials in humans with T2DM demonstrate that curcumin supplementation significantly reduces fasting glycaemia and glycosylated hemoglobin (HbA1c) [246]; it also improves insulin resistance while optimizing lipid profiles and vascular health markers [247]. Moreover, curcumin demonstrates significant therapeutic potential in the management of diabetes by improving glucose and lipid metabolism, enhancing insulin sensitivity, and reducing insulin resistance in experimental animal models of diabetes [247]. Studies demonstrate that curcumin exerts cytoprotective effects against oxidative stress by enhancing HO-1 activity in vascular endothelial cells and inducing the production of antioxidants (e.g., SOD, CAT, and GSH) and inducible nitric oxide synthase (iNOS) inhibitors [248,249]. Curcumin functions as a SIRT1 activator, upregulating both the expression and activity of SIRT1 to confer protection against metabolic and vascular damage. This protective effect is mediated through deacetylation of p53 and subsequent reduction in apoptosis [250]. Moreover, curcumin alleviates cardiac fibrosis and lipid metabolism abnormalities in diabetes by activating the AMPK/SIRT1 signaling pathway [244,250]; concurrently, it suppresses the production of pro-inflammatory cytokines and macrophage inflammatory protein-1α (MIP-1α), thereby attenuating inflammatory responses [251]. Substantial evidence demonstrates that curcumin prevents and treats diabetic complications through multi-target mechanisms, including amelioration of oxidative stress, suppression of pro-inflammatory pathways, and reduction in hepatic gluconeogenesis in diabetic patients.

Lignans are polyphenolic derivatives that are widely distributed in vascular plants and that can be isolated from various plant organs, including leaves, stems, xylem, roots, rhizomes, and resinous exudates. These compounds are particularly abundant in dietary sources such as legumes, whole grains, vegetables, fruits, and beverages. [245,252]. Lignans exhibit multiple bioactive properties, including antioxidant, anti-inflammatory, antitumor, and anti-neurodegenerative activities [252]. Research demonstrates that lignans significantly enhance glucose uptake and improve insulin sensitivity through activation of the PI3K/Akt signaling pathway [253]. Notably, multiple high-quality human randomized controlled trials (RCTs) have demonstrated that lignans—primarily secoisolariciresinol diglucoside (SDG) derived from flaxseed and silybin, administered at doses of 200–600 mg/day for from 8 weeks to 6 months—significantly improved key metabolic parameters in patients with T2DM. This included effective reductions in fasting blood glucose (FBG), HbA1c, total cholesterol (TC), and the key inflammatory cytokine TNF-α, alongside an increase in beneficial adiponectin levels [254,255,256,257]. Furthermore, lignans augment the activity of antioxidant enzymes, including SOD and CAT, thereby mitigating oxidative damage in diabetic tissues [258]. Beyond their antioxidant properties, lignans exhibit anti-inflammatory effects by suppressing the NF-κB pathway, which regulates pro-inflammatory cytokine expression, consequently reducing the levels of inflammatory mediators such as TNF-α and IL-6 [259]. The significant anti-inflammatory effects observed clinically (such as the reduction in TNF-α) corroborate the mechanism whereby lignans inhibit the NF-κB pathway. These findings substantiate the therapeutic potential of lignans in both the prevention and management of diabetes and its complications.

#### 5.2.2. Flavonoids

Flavonoids are a class of naturally occurring polyphenolic secondary metabolites and are among the most common polyphenolic compounds in the human diet [260]. They are further classified into six subclasses: flavonols, flavones, flavanones, isoflavones, flavanols, and anthocyanins [261]. Based on various in vitro, animal, and human studies, flavonoids may influence multiple metabolic processes in diabetes and help alleviate oxidative stress, stress-sensitive signaling pathways, and inflammatory responses [262].

Quercetin, a component of flavonols, is abundantly present in the human diet [261]. Studies have shown that quercetin can effectively prevent and ameliorate diabetic complications. Its beneficial effects are largely attributed to its anti-inflammatory and antioxidant properties. Molecular targets influenced by quercetin include TNF-α, NF-κB, AMPK, AKT, and Nrf2 [263]. Quercetin can enhance the expression of Nrf2 and promote its translocation to the nucleus, thereby contributing to the upregulation of antioxidant gene expression [264]. Meanwhile, quercetin can reduce the expression of iNOS by inhibiting inflammatory signaling pathways such as NF-κB, thereby decreasing the excessive production of nitric oxide (NO) and alleviating oxidative damage [264]. Quercetin reduces the release of pro-inflammatory cytokines such as TNF-α, IL-6, IL-1β, and IL-8 by inhibiting the expression of cyclooxygenase-2 (COX-2) [265]. Moreover, quercetin has been shown to promote the secretion of the anti-inflammatory cytokine IL-10. Clinical studies further confirm that quercetin supplementation (≥500 mg/day for ≥8 weeks) in humans significantly improves FBG, Homeostatic Model Assessment of Insulin Resistance (HOMA-IR), and levels of the aforementioned inflammatory markers (e.g., TNF-α and IL-6) in patients with T2DM and metabolic syndrome. Comparable metabolic improvements were also observed in patients with polycystic ovary syndrome (PCOS) [266,267,268,269,270]. Furthermore, quercetin enhances glucose uptake by activating the insulin-mediated MAPK signaling pathway and promotes the phosphorylation of the PI3K/Akt signaling cascade, providing a molecular basis for its antidiabetic properties [271]. Collectively, through its multi-target actions, quercetin enhances antioxidant defenses, mitigates inflammatory damage, and improves glucose metabolic dysregulation, demonstrating significant potential for the prevention and management of diabetes and its complications.

Rutin is a flavonoid compound that is found in many plants, commonly found in some fruits and peels, especially citrus fruits [272]. It has a wide range of biological activities, including anti-inflammatory, antioxidant, neuroprotective, renoprotective, myocardial protective, and hepatoprotective effects [273]. Studies have shown that rutin can exert antioxidant and anti-inflammatory effects by scavenging free radicals, inhibiting lipid peroxidation, and regulating inflammatory factors. In anti-diabetes treatment, rutin can reduce blood glucose, regulate insulin secretion, improve dyslipidemia, and activate IRS-2/PI3K/Akt/GSK-3β signaling pathways [6,274]. At the same time, it can also reduce the absorption of carbohydrates in the small intestine, inhibit gluconeogenesis, promote tissue glucose uptake, enhance β-cell insulin secretion, and protect islet structure. Rutin can also inhibit the production of AGEs and their precursors, sorbitol, and oxidative stress products, representing a potential protective effect against the kidney, nerve, liver, and cardiovascular complications caused by diabetes [273]. Notably, clinical evidence confirms that 60-day rutin supplementation in diabetic patients significantly reduces the concentrations of renal function markers—specifically serum urea and creatinine—with levels reverting upon treatment discontinuation, validating its dose-dependent renoprotective effects [273].

Proanthocyanidins (PCs) are a kind of flavonoids, with catechols or epicatechols as fundamental components, that are widely found in plants, commonly including grape seed extract or French coast pine bark extract [275,276]. PCs have a variety of biological activities, such as anti-obesity, anti-diabetes, anticancer, anti-inflammatory, antioxidant, and cardiovascular protection effects [277]. Studies have shown that PCs alleviate oxidative stress by activating the AMPK and Nrf2 pathways, increase antioxidants such as SOD and GSH, and reduce inflammatory responses mediated by NF-κB and NLRP3 [278,279,280,281]. PCs promoted the transformation of pro-inflammatory M1 macrophages to anti-inflammatory M2 macrophages, which was marked by increased expression of Arg1, Ym1, and Fizz1 [282]. Clinical evidence confirms that a 1:1 combination formula containing epigallocatechin-3-gallate (EGCG) and *Emblica officinalis* significantly enhances antioxidant defenses and synergistically improves clinical outcomes in patients with diabetic nephropathy [283]. In addition, PCs also play a key role in the prevention and control of diabetes and its complications by regulating cytokines such as IL-1β, TNF-α, and IL-12 to reduce the inflammatory response [277].

#### 5.2.3. Terpenoids

Terpenoids are classified into monoterpenes, sesquiterpenes, diterpenes, triterpenes, tetraterpenes, and polyterpenes, based on the number of isoprene units in their molecular structure, with triterpenoids being the most prevalent group [284]. Triterpenoids are ubiquitously distributed in nature, existing either in free forms or as glycosides and esters, with predominant occurrence in leaves, stem barks, fruits, and roots [284,285]. Triterpenoids exhibit extensive pharmacological properties and significant bioactivities, including anticancer, antioxidant, anti-inflammatory, anti-atherosclerotic, and antiviral effects [286]. Research has demonstrated that these compounds possess multiple antidiabetic mechanisms: they can inhibit key enzymes involved in glucose metabolism, prevent the development of insulin resistance, and normalize blood glucose and insulin levels. Compared to synthetic drugs, these natural compounds not only demonstrate hypoglycemic effects but also exhibit hypolipidemic and anti-obesity activities [287]. Moreover, triterpenoids demonstrate potent antioxidant activity and effectively inhibit the formation of AGEs, positioning them as promising therapeutic agents for preventing and treating diabetic complications [287,288]. For example, oleanolic acid and ursolic acid, as potent antioxidants, significantly modulate the production of AGEs in renal tissues. Treatment with these compounds has been shown to suppress the formation of renal AGEs and improve kidney function in streptozotocin (STZ)-induced diabetic mice [289]. Clinical studies demonstrate that terpenoid extracts (400–900 mg/day for ≥8 weeks) significantly improve metabolic parameters in T2DM patients: an andrographolide-containing formulation reduced FBG and uric acid; fenugreek seed extract substantially decreased HbA1c; and caper fruit extract effectively lowered HbA1c and FBG while improving hepatic and renal markers. Furthermore, a 12-week intervention with the pentacyclic triterpenoid ursolic acid significantly enhanced insulin sensitivity and suppressed lipogenesis [290,291,292,293,294].

#### 5.2.4. Alkaloids

Alkaloids represent a major class of nitrogen-containing phytochemicals that are widely distributed throughout the plant kingdom. These compounds exhibit a broad spectrum of biological and pharmacological properties, including well-documented antidiabetic effects [295]. Alkaloids mediate their therapeutic effects on glycemic pathology through diverse signaling cascades and pathways, including inhibition of α-glucosidase activity, blockade of protein tyrosine phosphatase 1B (PTP-1B), inactivation of dipeptidyl peptidase-IV (DPP-IV), enhancement of insulin sensitivity, and modulation of oxidative stress [296]. These coordinated multi-target actions collectively ameliorate diabetes and its complications. Certain monomeric alkaloids—most notably berberine and capsaicin—have garnered significant attention in this field.

Berberine, an isoquinoline alkaloid derived from medicinal herbs such as Coptis, Phellodendron, and Berberis [297], exhibits multiple pharmacological activities, including antihyperglycemic, antioxidant, anti-inflammatory, and anti-apoptotic effects [298]. Research has demonstrated that berberine administration significantly reduces FBG, HbA1c, pro-inflammatory cytokine levels, and oxidative stress markers [299]. Moreover, berberine stimulates insulin secretion and ameliorates insulin resistance through multiple pathways, demonstrating protective effects against diabetes complications [298]. Notably, berberine attenuates hepatic insulin resistance through the miR-146b/SIRT1 pathway, representing a novel therapeutic target for metabolic diseases [300].

Capsaicin, the primary pungent constituent of chili peppers, exhibits diverse biological activities, including anti-inflammatory, antioxidant, anti-obesity, and antitumor properties, and has been increasingly utilized as an active pharmaceutical ingredient in recent years [301]. Capsaicin has been shown to inhibit non-enzymatic glycation and reduce the accumulation of AGEs, thereby alleviating glycative stress in diabetic patients. This mechanism effectively prevents and treats AGE-induced diabetic complications [302].

**Table 1:** Natural products targeting diabetic complications: sources, core mechanisms, and representative compounds.

Chemical Class	Representative Compounds	Primary Sources	Core Mechanisms	References
Polyphenols	Resveratrol	Cereals, fruits, vegetables, legumes	- Activates SIRT1/FOXO pathway - Inhibits NOX2/4 - Induces HO-1 expression - Suppresses NF-κB	[234,235,236,237,238,239,240,241]
	Curcumin	Turmeric rhizomes	- Enhances HO-1 activity - Induces SOD/CAT/GSH - Inhibits MIP-1α/TNF-α	[243,244,247,248,249,251]
	Lignans	Flaxseeds, whole grains	- Activates PI3K/AKT pathway - Boosts SOD/CAT activity - Inhibits NF-κB	[245,252,253,258,259]
Flavonoids	Quercetin	Onions, apples, citrus fruits	- Promotes Nrf2 nuclear translocation - Inhibits COX-2/iNOS - Increases IL-10 secretion	[261,262,263,264,265,271]
	Rutin	Citrus peel	- Activates IRS-2/PI3K/AKT pathway - Inhibits AGE formation - Scavenges free radicals	[6,272,273,274]
	Proanthocyanidins	Grape seeds, pine bark	- Activates AMPK/Nrf2 - Promotes M2 macrophage polarization - Inhibits NLRP3 inflammasome	[275,277,278,279,280,281,282]
Terpenoids	Oleanolic acid/ursolic acid	Olive leaves, rosemary, apple peel	- Inhibits α-glucosidase - Blocks AGE formation - Activates Nrf2 pathway	[284,285,286,287,288,289]
Alkaloids	Berberine	Coptis, Phellodendron	- Inhibits DPP-IV/α-glucosidase - Reduces FBG/HbA1c - Modulates gut microbiota	[295,296,297,298,299]
	Capsaicin	Chili peppers	- Inhibits non-enzymatic glycation - Reduces AGE accumulation - Activates TRPV1 receptor	[301,302]

### 5.3. Epigenetic Targeting by Natural Therapeutics

Epigenetic regulation influences an individual’s response to natural therapies. This is primarily manifested as various natural compounds directly targeting and modifying key epigenetic mechanisms, such as DNA methylation and histone modifications, to regulate gene expression and cellular functions [303]. For example, resveratrol activates the NAD-dependent histone deacetylase SIRT1, improving glucose homeostasis and insulin sensitivity [304,305]. Curcumin inhibits histone acetyltransferases (HATs), histone deacetylases (HDACs), and DNA methyltransferases (DNMTs) and influences miRNA activity [306]. Quercetin promotes histone H3 acetylation, potentially inhibits HDACs, and stimulates glucose uptake through an MAPK-dependent mechanism [307,308,309,310]. Other natural compounds, such as EGCG, enhance the anti-inflammatory activity of regulatory T cells. This is achieved by modulating histone acetylation/deacetylation and DNA methylation [311]. Sulforaphane inhibits DNMT1 expression, reduces promoter methylation, and suppresses HDACs [312,313,314]. Genistein reverses hypermethylation and induces active histone modifications [315,316]. These epigenetic alterations regulate key pathophysiological processes. Specifically, they improve insulin resistance, protect β-cell function, suppress inflammation, and modulate gluconeogenesis and glucose production. Consequently, they mediate the therapeutic efficacy of natural therapies against diabetes and its complications; they also contribute to natural therapies’ effects against cancer [317]. An individual’s response to these natural therapies depends on their ability to intervene in specific epigenetic targets and pathways.

### 5.4. Barriers to Clinical Deployment of Natural Therapies

#### 5.4.1. Preclinical–Clinical Efficacy Discrepancies

Despite exhibiting distinct advantages in multi-target modulation for preventing and treating diabetic complications, natural compounds face formidable challenges in clinical translation. A central manifestation of this limitation is the frequent failure to translate the robust therapeutic efficacy observed in preclinical models into consistent clinical outcomes in humans. For example, curcumin demonstrates robust hepatorenal repair and wound-healing promotion in animal models. However, human trials reveal only partial improvements in specific inflammatory markers such as C-reactive protein (CRP) [318,319,320,321,322,323,324,325]. Lignans exhibit insulin-sensitizing effects in preclinical studies; however, in human applications, their glucose-lowering effects are weak and inconsistent [254]. In addition, clinical studies on hesperidin—a flavonoid compound—have reported conflicting outcomes. A meta-analysis investigating the effects of hesperidin supplementation on glycemic control markers found no significant impacts on FBG, insulin, HbA1c, HOMA-IR, or the Quantitative Insulin Sensitivity Check Index (QUICKI) [326]. However, these findings directly contradict a recent systematic review examining dietary polyphenols’ influence on metabolic syndrome features, which proposed beneficial effects of hesperidin on glucose metabolism and insulin resistance [327]. These discrepancies underscore critical limitations in extrapolating preclinical findings to human therapeutics.

#### 5.4.2. Patient Heterogeneity Impacts

In the clinical translation process, patient heterogeneity and comorbidities constitute key determinants that complicate the clinical translation of natural compounds. Divergent responses arise from differences in diabetes stages. For instance, curcumin may protect pancreatic β-cells and delay disease onset in prediabetic individuals, yet it exhibits variable glucose-lowering efficacy in diagnosed patients [318,319,320,321,322,323,324,325]. The extent of this variability is closely correlated with the severity of obesity and insulin resistance status. For instance, the oral absorption of lignan compounds is influenced by gut microbiota composition and genetic variability, leading to significant inter-individual variations in circulating metabolite profiles [328,329,330]. Concurrently, the nature of comorbid conditions can redirect core therapeutic priorities. In patients with metabolic syndrome, curcumin’s primary clinical benefit manifests as lipid regulation—specifically elevating high-density lipoprotein while reducing low-density lipoprotein [318,331]. In patients with comorbid cardiovascular disease, the drug’s anti-inflammatory and antioxidant properties, manifested in reductions in CRP, become a particularly valuable benefit [332]. Furthermore, concomitant medication regimens represent critical heterogeneity factors. Early studies suggest that cocoa products rich in flavonoids may contribute to cardiovascular protection. However, supplementation with cocoa flavanols in type 2 diabetic patients receiving stable antihypertensive therapy failed to improve cardiometabolic parameters. This occurred because antihypertensive drugs and natural constituents may partially modulate shared biological targets, compromising their synergistic efficacy [333]. This observation suggests that such interventions hold greater preventive potential for treatment-naïve individuals with prediabetes or early metabolic dysregulation. Collectively, these factors constitute core dimensions of complexity in the clinical deployment of natural compounds.

#### 5.4.3. Clinical Limitations of Natural Compounds

In addition to the clinical barriers described above, natural compounds still face numerous significant limitations in clinical application. The foremost challenge lies in their low bioavailability. Many active components exhibit poor intestinal mucosal penetration due to complex chemical structures and undergo rapid metabolic deactivation during hepatic first-pass effects, resulting in insufficient therapeutic concentrations in vivo [334]. Beyond pharmacokinetic hurdles, safety concerns demand rigorous evaluation. For instance, long-term quercetin intake carries potential mutagenicity risks [333,335]. Curcumin administration may induce mild gastrointestinal adverse effects [246]. Another major limitation stems from insufficient mechanistic elucidation, complicating the attribution of therapeutic efficacy. Natural compounds often concurrently target multiple signaling pathways, such as AMPK and NF-κB, complicating the attribution of observed therapeutic benefits to individual pathways versus synergistic interactions. Compounding these issues is the scarcity of large-scale clinical trials. Due to compositional complexity and standardization challenges, most natural products lack systematic assessment of their long-term safety profiles, including potential risks such as genotoxicity and organ damage. Although certain natural products have demonstrated improvements in glycemic control or complication alleviation in small-scale preliminary studies, rigorous validation of their efficacy and safety in broader populations remains imperative [334]. Thus, overcoming bioavailability barriers, establishing safety profiles, clarifying mechanisms of action, and generating robust clinical evidence are essential for successful clinical translation.

## 6. Conclusions and Perspectives

As we know, controlling the onset and progression of diabetic complications is a global challenge. The pathogenesis of diabetic vascular complications is exceptionally complex, and we still lack a comprehensive understanding and adequate therapies to address the projected rise in incidence. In this review, we emphasize that these complications arise from an intertwined oxidative–inflammatory network. We systematically describe how chronic hyperglycemia drives pathological processes through the interplay of metabolic dysregulation, mitochondrial dysfunction, ER stress, and epigenetic modulation. Together, these factors promote oxidative stress and inflammation and ultimately lead to multi-organ vascular damage. Natural multi-target agents have unique advantages in this context. Unlike drugs that act on a single pathway, compounds such as resveratrol, quercetin, curcumin, and berberine can simultaneously activate Nrf2-mediated antioxidant defenses, inhibit NF-κB signaling and inflammasome activation, and modulate epigenetic regulators. As a result, they often provide more pronounced protection—enhancing antioxidant capacity, reducing inflammatory markers, and repairing tissue pathology—than glucose-lowering therapies alone. This multi-faceted approach offers diversified therapeutic options for diabetic vascular complications.

Despite their promising mechanisms and broad potential, clinical translation of natural products remains hindered by challenges such as low bioavailability, variable potency, patient heterogeneity, and a lack of large-scale clinical trials. We anticipate that future research teams will continue to discover new natural bioactive compounds and investigate their precise mechanisms of action. Elucidating pharmacological properties and rigorously evaluating clinical applications will be essential to provide safer and more effective treatments for patients. Moreover, designing clinical studies that emphasize long-term efficacy and safety in combination therapies is crucial. Given the heterogeneity of diabetes, exploring treatment regimens tailored to more refined patient subgroups will deepen our understanding of individual responses and guide more effective clinical management.
